# Remodeling of an *in vitro* microvessel exposed to cyclic mechanical stretch

**DOI:** 10.1063/5.0010159

**Published:** 2021-04-02

**Authors:** Soheila Zeinali, Emily K. Thompson, Holger Gerhardt, Thomas Geiser, Olivier T. Guenat

**Affiliations:** 1 Organs-on-Chip Technologies Laboratory, ARTORG Center, University of Bern, 3008 Bern, Switzerland; 2 Integrative Vascular Biology Laboratory, Max-Delbrück Center for Molecular Medicine in the Helmholtz Association (MDC),13092 Berlin, Germany; 3 Department for BioMedical Research, University of Bern, 3008 Bern, Switzerland; 4 Department of Pulmonary Medicine, Inselspital, University Hospital of Bern, 3010 Bern, Switzerland; 5 Division of General Thoracic Surgery, Inselspital, University Hospital of Bern, 3010 Bern, Switzerland

## Abstract

In the lungs, vascular endothelial cells experience cyclic mechanical strain resulting from rhythmic breathing motions and intraluminal blood pressure. Mechanical stress creates evident physiological, morphological, biochemical, and gene expression changes in vascular endothelial cells. However, the exact mechanisms of the mechanical signal transduction into biological responses remain to be clarified. Besides, the level of mechanical stress is difficult to determine due to the complexity of the local distension patterns in the lungs and thus assumed to be the same as the one acting on the alveolar epithelium. Existing *in vitro* models used to investigate the effect of mechanical stretch on endothelial cells are usually limited to two-dimensional (2D) cell culture platforms, which poorly mimic the typical three-dimensional structure of the vessels. Therefore, the development of an advanced *in vitro* vasculature model that closely mimics the dynamic of the human lung vasculatures is highly needed. Here, we present the first study that investigates the interplay of the three-dimensional (3D) mechanical cyclic stretch and its magnitude with vascular endothelial growth factor (VEGF) stimulation on a 3D perfusable vasculature *in vitro*. We studied the effects of the cyclic strain on a perfusable 3D vasculature, made of either human lung microvascular endothelial cells or human umbilical vein endothelial cells embedded in a gel layer. The *in vitro* 3D vessels underwent both *in vivo*-like longitudinal and circumferential deformations, simultaneously. Our results showed that the responses of the human lung microvascular endothelial cells and human umbilical vein endothelial cells to cyclic stretch were in good agreement. Although our 3D model was in agreement with the 2D model in predicting a cytoskeletal remodeling in response to different magnitudes of cyclic stretch, however, we observed several phenomena in the 3D model that the 2D model was unable to predict. Angiogenic sprouting induced by VEGF decreased significantly in the presence of cyclic stretch. Similarly, while treatment with VEGF increased vascular permeability, the cyclic stretch restored vascular barrier tightness and significantly decreased vascular permeability. One of the major findings of this study was that a 3D microvasculature can be exposed to a much higher mechanical cyclic stress level than reported in the literature without any dysfunction of its barrier. For higher magnitudes of the cyclic stretch, the applied longitudinal strain level was 14% and the associated circumferential strain reached the equivalent of 63%. In sharp contrast to our findings, such strain typically leads to the disruption of the endothelial barrier in a 2D stretching assay and is considered pathological. This highlights the importance of 3D modeling to investigate mechanobiology effects rather than using a simple endothelial monolayer, which truly recapitulates the *in vivo* situation.

## INTRODUCTION

The lung microvasculature is continuously exposed to mechanical cyclic stretch (CS) induced by the rhythmic breathing motions and the shear stress generated by the blood flow. These mechanical cues act on the vascular walls and play an important role in the endothelial barrier remodeling. Small pulmonary capillaries are particularly exposed to lung expansion and contraction of the respiratory cycle. During inhalation, extra-alveolar capillaries, located outside of the alveolar region, stretched and have an increased cross-sectional area.^1^ However, alveoli capillaries embedded in the air-blood barrier are compressed between two alveoli. During exhalation, the opposite occurs.^1^ The direct measurement of the vascular strain in the lung is difficult and has not yet been reported.^2^ Tschumperlin *et al.* correlated the lung expansion during breathing with an increase in the epithelial cell surface.^3,4^ Although the extent of the stretch that pulmonary microvascular cells undergo is not determined yet, *in vitro* studies aimed at investigating endothelial cell mechanotransduction have adopted epithelial stretch values.^5–7^

Endothelial mechanosensors sense and transduce cyclic strain stimuli into biological signals. Although stretch-activated ion channels, integrins, and platelet endothelial cell adhesion molecule-1 (PECAM-1) are well-known endothelial mechanosensors, the precise mechanism of the overall endothelial mechanotransduction is not yet completely understood.^4,8–11^ Mechanical forces at the surface of endothelial cells cause tyrosine phosphorylation of PECAM-1 and phosphorylation of extracellular signal-regulated kinase.^9,11–13^ PECAM-1 is localized at the endothelial cell junctions and becomes mechanosensitive when the endothelial barrier is confluent.^9^ Integrins are transmembrane cell-matrix receptors that undergo structural and functional changes in response to mechanical force.^10,14^ Although the downstream signaling pathways of integrins have been characterized, the original molecular cascades that link integrins to these pathways need to be elucidated. Uniaxial stretching of human pulmonary microvascular endothelial cells increases the intracellular Ca^2+^ concentration in a strain amplitude-dependent manner for strains between 10% and 30%.^12^ This finding is important as irregular Ca^2+^ homeostasis caused by mechanical stretch during ventilation may play a role in the progression of acute lung injury.^15^ In addition to mechanical factors, chemical signaling can induce changes in the integrin cytoplasmic domains. Vascular endothelial growth factor (VEGF) is, for instance, a fundamental regulator of vascular remodeling.^16^ VEGF is specific to endothelial cells and plays a unique role in preventing their apoptosis and controlling the physiological and pathological growth of blood vessels.^17^ Because of its ability to induce vascular leakage, VEGF is also known as “vascular permeability factor.”^17^ These leakages take place during the creation of vascular sprouting. VEGF disrupts cell–cell adhesion, directs tip cell migration, determines stalk cell proliferation, and promotes endothelial cell sprouting in the surrounding tissue.^18,19^ The inhibition of VEGF downregulates vascular sprouting and angiogenesis.^20^ The effect of 3D CS and the interplay between CS and VEGF in regulating vascular remodeling and angiogenesis are still not fully understood and require sophisticated *in vitro* models that are able to integrate both features.

Recently, microvasculature-on-chips gained interest to investigate pathophysiological situations involving vessels.^21^ Among different techniques, two strategies are common to develop these models. The first technique is based on the self-assembly of endothelial cells with or without mural cells to form functional three-dimensional (3D) microvessels in a hydrogel matrix.^22–25^ This technique replicates the physiological processes taking place during vasculogenesis and angiogenesis and leads to the formation of an interconnected 3D microvascular network. The second strategy is based on the creation of a vascular-like pattern made of a sacrificial material.^26–28^ Once removed, the artificially generated tubes are perfused and seeded with endothelial cells, which then populate the inner tube surface and form a confluent endothelial layer.^29–32^ Microvasculature-on-chips have been used to study drug toxicity and efficacy^23,33^ and to investigate the re-localization of tumor cells through intra- and extravasation,^34^ vascular disease modeling,^35^ and endothelial mechanotransduction.^36^

So far, the effect of the cyclic mechanical stress on the endothelial barrier has been mostly based on 2D cell culture platforms. These platforms consist of a stretchable polymeric foil on which the cells are cultured and the foil can be stretched uniaxially or biaxially.^6,7,9,16,37,38^ Birukov and colleagues exposed human pulmonary artery endothelial cells cultured on such membranes to different magnitudes of uniform radial and circumferential planar strains. They observed that thrombin-induced gap formation on the endothelial monolayer is dependent on the magnitude of CS.^38^ The preconditioning monolayer of human lung arterial endothelial cells with pathological magnitude of planar CS (18%) significantly increased the gap surface area induced by thrombin compared to the static condition.^38^

Due to the complex distribution of the strains in the lung parenchyma, the direct measurement of vascular strain in the lungs has not yet been reported. Nevertheless, one usually assumes that the level of CS to which the lung microvasculature is exposed to is similar to the stress of the alveolar epithelium.^4^ *In vitro* studies of alveolar epithelial cells exposed to 2D CS reported a 25% increase in the cell surface area, which is equivalent to an 8%–12% linear strain. This corresponds to a physiological CS, whereas a 37%–50% increase in the cell surface area is equivalent to a 17%–22% linear strain and is related to a pathological CS.^3,38,39^ Stretchable monolayers of endothelial cells are useful to answer fundamental questions of endothelial mechanotransduction. However, they are unable to mimic the 3D CS experienced by vessels from cyclically expanding organs, such as the lungs, and, thus, not suited for the study of the cyclic stretch effect on 3D microvessels.

The recent development of perfusable 3D microvasculature^22,23,25,40^ is a milestone in vascular endothelial cell biology, as these models much better recapitulate the *in vivo* situation than monolayers of endothelial cells. They have the potential to provide a new level of understanding of mechanobiology effects. Several of those models aimed at investigating the effects of the shear stress generated by the blood flow.^36,41,42^ Very recently, Shimizu *et al.* reported about the effect of uniaxial strain on gelatin-based 3D microchannels made of human umbilical vein endothelial cells (HUVECs).^43^ In this study, simultaneous mechanical stimulation of the extra cellular matrix (ECM)-based vasculature was developed based on the 3D culture system of the skin equivalent capable of perfusion and stretching stimuli.^43^ They showed that the combination of the fluid shear stress and uniaxial mechanical stretch aligned HUVECs with the flow direction and the vertical direction of the stretching motion.^43^ This model would be suitable to simulate the cyclic strain generated in straight arteries.^44^ However, a uniaxial strain does not accurately mimic the nature of the three-dimensional deflection experienced by the pulmonary vasculature. To do so, a model able to induce simultaneous circumferential and longitudinal strains on the vasculature is needed.

In this study, we propose for the first time a dynamic microvasculature platform aimed at investigating the effect of 3D CS on human vascular remodeling. The platform incorporates a 200 um diameter 3D perfusable microvasculature located in the middle of a fibrin gel layer, which is cyclically deflected. The vasculature exposed to this 3D CS simultaneously experiences longitudinal and circumferential strains. Functional vasculatures made of human umbilical vein endothelial cells (HUVECs) or human lung microvasculature cells (VeraVecs) are reproduced and exposed to 3D cyclic mechanical deflection. Most of the experiments were carried out with primary HUVEC cells, easier to culture than lung microvascular endothelial cells, and some key experiments were reproduced with the Veravecs, exposed *in vivo* to the cyclic mechanical stress of the respiratory movements. Additionally, the effects of different magnitudes of the CS—physiological (low) and pathological (high)—and the interplay between the CS and VEGF stimulation are investigated in terms of vascular permeability, vascular sprouting, vascular morphology, cellular elongation, and cellular polarization.

## RESULTS

### Longitudinal and circumferential strains of vasculature *in vitro*

**FIG. 1.**
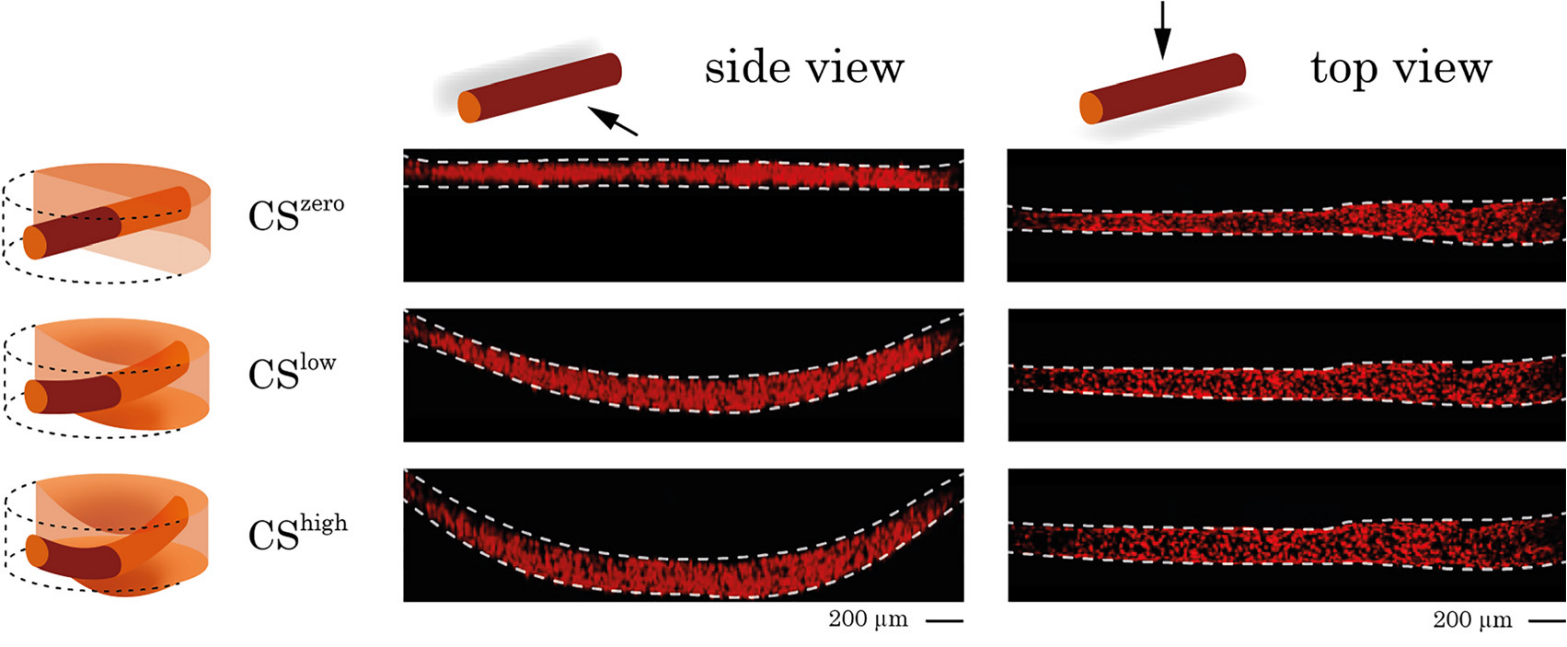
Side and top views of a 3D vessel under CS^zero^, CS^low^, and CS^high^ conditions. RFP-labeled endothelial cells were cultured in the chip and formed a 3D vessel. The vessel is visualized in the static condition and under different magnitudes of stretch using confocal microscopy. Side and top views of the vessel were used to measure the vessel length and diameter and to quantify the longitudinal and circumferential strains.

A 200 *μ*m diameter cylindrical channel was created within a 500 *μ*m thick fibrin gel layer supported by a polydimethylsiloxane (PDMS) membrane. RFP-labeled VeraVecs were introduced in the channel to populate the vessel walls until the 3D confluent endothelial barrier in the chip is reached. The assembly comprising the membrane, the gel layer, and the vasculature was cyclically stretched in 3D by applying a cyclic negative pressure in the chamber located beneath the membrane via a house-made electro-pneumatic pump (breather). Figure 1 shows the top and side view of a 3D vessel in static (CS^zero^) and stretch (CS^low^ and CS^high^) conditions. These images illustrate the deflection and expansion of the vasculature under stretch and were used to determine the vascular length and diameter. From these measurements, the longitudinal and circumferential strains of the vessels were calculated using the below equations:

$${\mathrm{Circumferential}\;\mathrm{Strain}\;(\epsilon }_{r}):\;\frac{r_{f}-r_{i}}{r_{i}}\times 100,$$

$${\mathrm{Longitudinal}\;\mathrm{Strain}\;(\epsilon }_{L}):\;\frac{L_{f}-L_{i}}{L_{i}}\times 100.$$

**FIG. 2.**
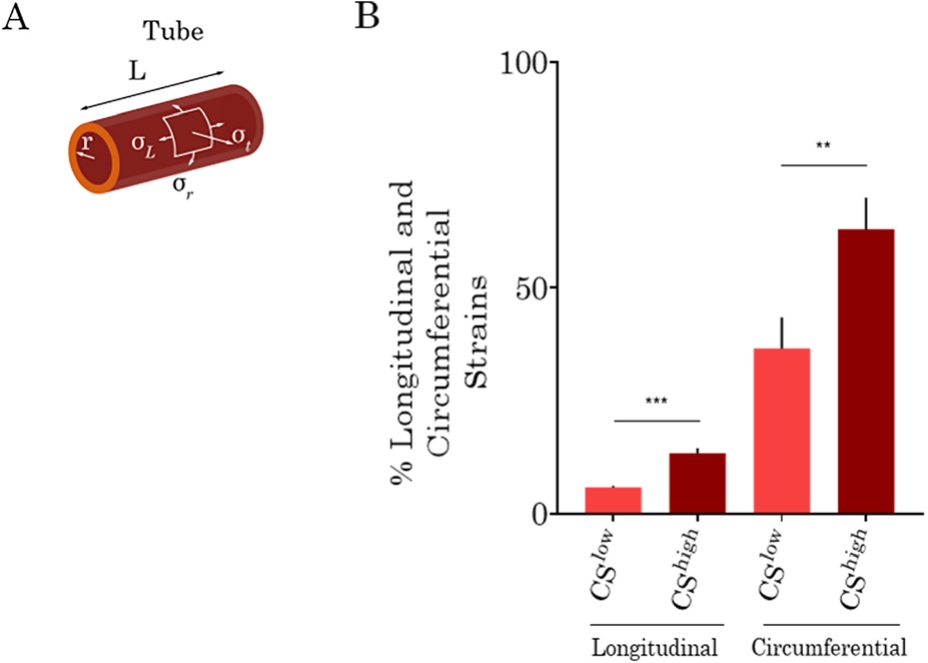
(a) Schematic of a 3D vessel with radius *r* and length *L* to show the induced strains upon 3D stretch. A longitudinal strain induces an elongation of the vessel, *σ_L_*, and a circumferential strain, *σ_T_*, increases the vessel perimeter. (b) Longitudinal and circumferential strains of three different vasculatures formed by VeraVecs under low and high magnitudes of cyclic stretch.

In these calculations, $r_{i}$ is the vessel radius before stretch, $r_{f}$ is the vessel radius under stretch, $l_{i}$ is the vessel length before stretch, and $l_{f}$ is the vessel length under stretch. As shown in Fig. 2(a), the longitudinal strain induces an elongation of the 3D vessel, whereas the circumferential strain increases the vessel perimeter. The radial strain, which acts on the endothelial walls, was not considered as it is negligible. Figure 2(b) shows the values of the vasculature strains under different magnitudes of mechanical deflection. The plots reflect longitudinal and circumferential strains of three different *in vitro* vessels exposed to different magnitudes of CS and confirm that circumferential strain is dominant for both levels of the C^low^ and CS^high^. Finite element modeling of the vasculature under mechanical stretch was performed using COMSOL. Longitudinal and circumferential displacement fields of the vasculature under CS^high^ stretch were used to quantify the longitudinal (8%) and circumferential (63%) strains as a result of simulation (supplementary material, Fig. S1).

### Cyclic stretch increases the cell length and surface area

**FIG. 3.**
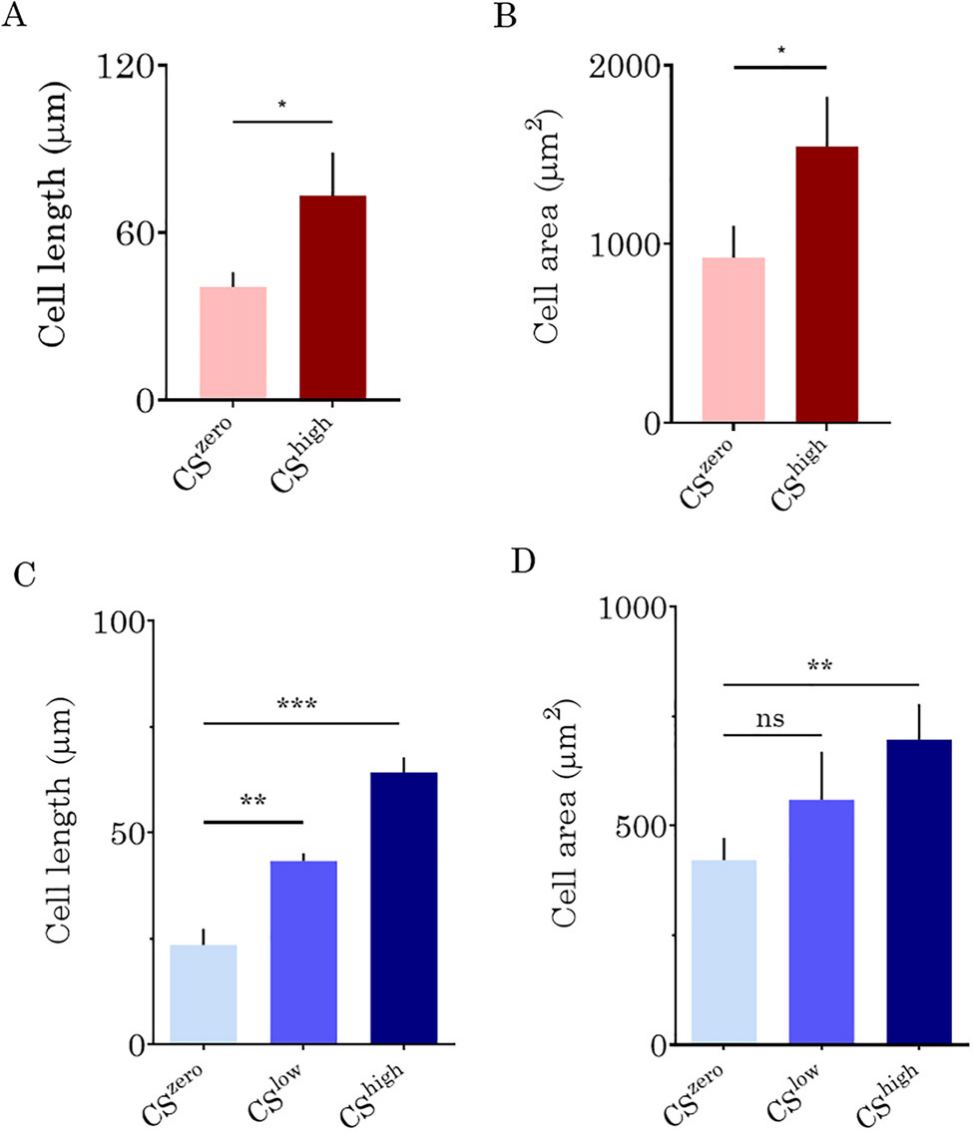
The lengths (a) and surface areas (b) of lung microvascular endothelial cells (VeraVecs) exposed to CS^high^, quantified using Fiji software. The cell length reflects the diagonal length of the cell. The effects of CS^low^ and CS^high^ on the HUVEC length (C) and surface areas (d).

*In vitro* vasculatures reproduced either by human lung microvascular endothelial cells (VeraVecs) or by human umbilical vein endothelial cells (HUVECs) respond to cyclic mechanical deflection by increasing their cell surface area and elongating. VeraVecs exposed to CS^high^ for 24 h showed a significant increase in the cell surface area (50%) and length (80%) compared to no stress condition [Figs. 3(a) and 3(b)].

Similarly, *in vitro* vasculature formed by HUVECs responds to cyclic mechanical deflection by the increased cell surface area and length [Figs. 3(c) and 3(d)]. When exposed to CS^high^, the cell surface area increases by 43% compared to the static condition. At CS^low^, the HUVEC surface area increases by 22%, which is, however, not significant compared to the cell surface area in the static condition (CS^zero^).

### Cyclic stretch regulates vascular morphology

**FIG. 4.**
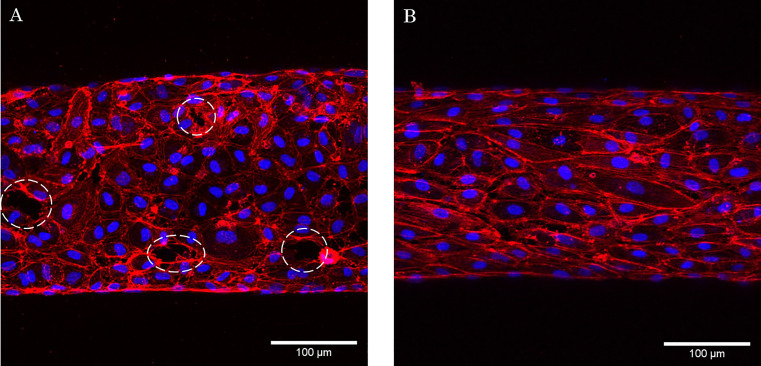
The effects of CS^high^ on the morphology of the vasculatures formed by VeraVecs. (a) Static condition and (b) exposed to CS^high^. Images are overlay of the F-actin (red) and DAPI (4′,6-diamidino-2-phenylindole) (blue) staining. CS^high^ resulted in cellular elongation and increasing vascular integrity. White arrows show the locations of the gaps on the vessel.

In the static condition, VeraVecs generated a confluent vasculature layer with a few gaps and with cells randomly oriented on the vessel wall. However, under CS^high^, the cells aligned in the direction of the vessel and elongated. The gaps observed in the static condition disappeared after being exposed to CS^high^, which resulted in an increased vascular integrity (Fig. 4).

**FIG. 5.**
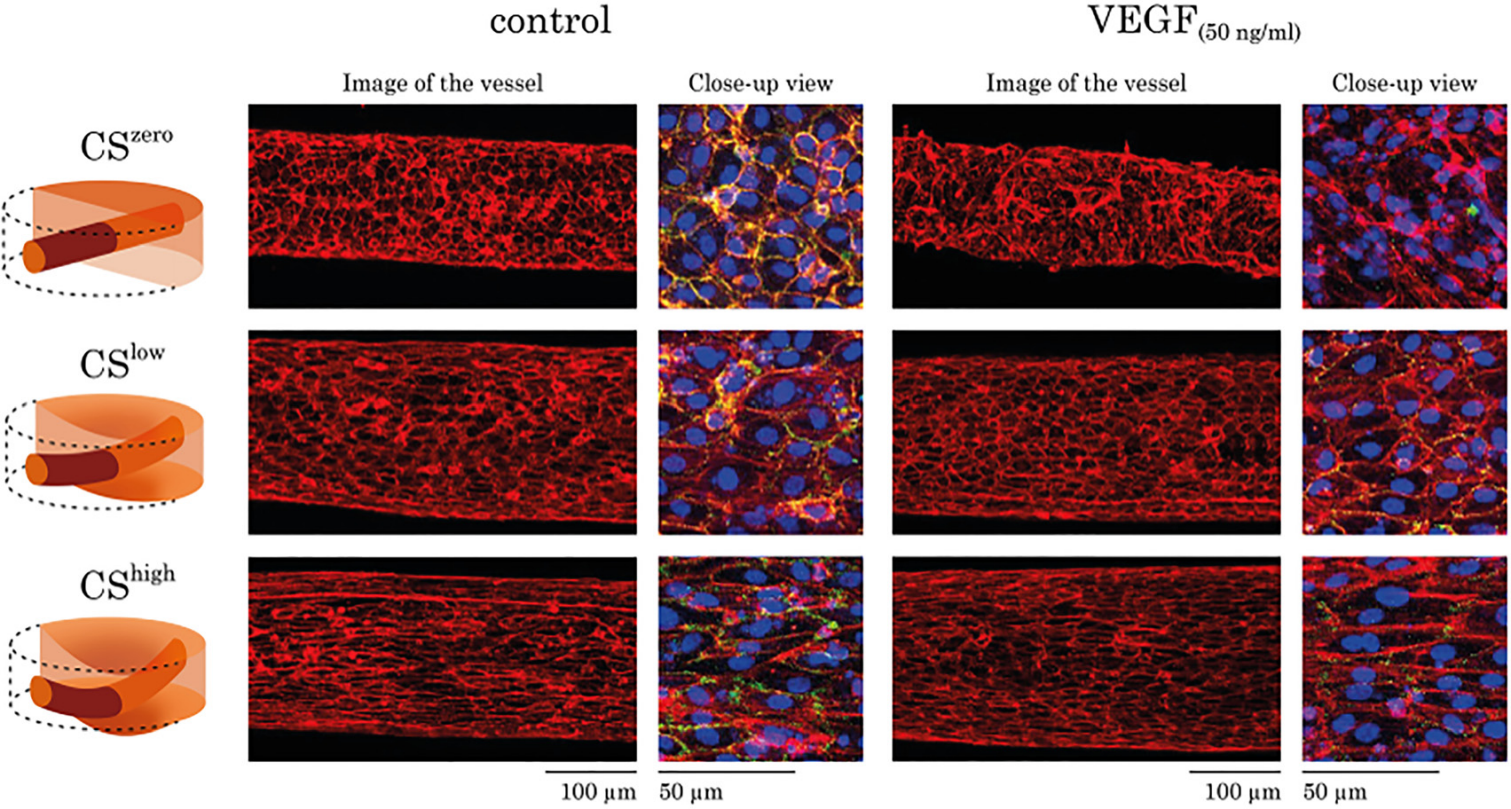
The effects of CS and VEGF (50 ng/ml) on morphology of the vasculature formed by HUVECs. For each experimental condition, images of the whole vessel showing F-actin staining are shown. For better visualization of the vascular morphology, the overlay of the F-actin (red), PECAM-1 (green), and DAPI (blue) staining is shown as a close-up view. Regardless of the treatment with VEGF, CS resulted in HUVEC actin re-organization, cellular elongation, and increasing vascular stability.

Besides, we examined the effects of CS^low^ and CS^high^ as well as VEGF (50 ng/ml) on the morphology of the vasculature formed by HUVECs. At CS^zero^, HUVECs produced a confluent vascular barrier, but they were randomly oriented in the vasculature wall. CS^low^ affected cellular orientation within the vascular wall and resulted in actin reorganization and cell elongation. As expected, CS^high^ increased these effects even more, by regulating vascular morphology and causing noticeable elongation of the cells on the vasculature wall. The addition of VEGF (50 ng/ml) in the fibrin gel reduced vascular stability and induced vascular sprouting. However, the exposure to CS^low^ normalized the distribution of the HUVECs on the vessel wall and preserved the stability of the VEGF-treated vasculature. When the mechanical stretch was further increased (CS^high^), the vascular stability was further enhanced, and also, the cells elongated significantly (Fig. 5). Under control and VEGF-treated conditions, CS-induced a cytoskeletal remodeling on endothelial cells.

### Cyclic stretch upregulates endothelial adhesion molecules

**FIG. 6.**
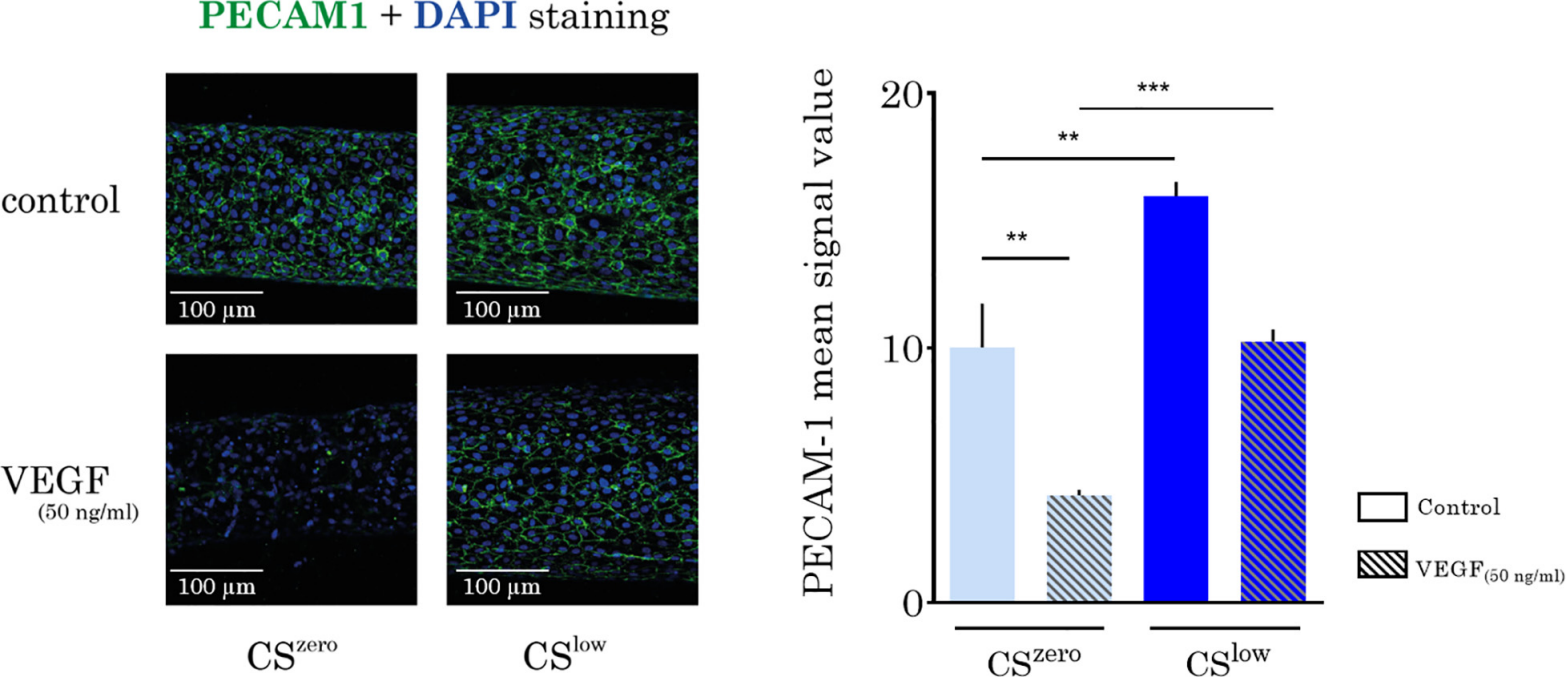
Cyclic stretch upregulates the platelet endothelial cell adhesion molecule (PECAM-1). Left: representative images show a section of the vessel formed by HUVECs for DAPI (blue) and PECAM-1 (green) staining in different experimental conditions. Images are Z-projection of the z-stacks from blue and green channels. Right: comparison of PECAM-1 levels from different experimental conditions; the mean signal value of the PECAM1 was quantified in Fiji. Treatment of the HUVECs with VEGF downregulated PECAM-1, and independent of the treatment, CS^low^ significantly increased PECAM-1 in the cell–cell junction.

To study the effect of CS on the levels of PECAM-1, vasculatures formed by HUVECs were immunostained against PECAM-1 and analyzed. Different magnitudes of the CS affect the levels of PECAM-1 in cell–cell junctions. As expected, when treated with 50 ng/ml of VEGF, endothelial cells considerably reduced vascular stability by downregulating PECAM-1. Our analysis of the different immunostained samples for PECAM-1 showed that regardless of the treatment with VEGF, even CS^low^ remarkably increased the level of PECAM-1 (Fig. 6).

### Cyclic stretch decreases vascular permeability

**FIG. 7.**
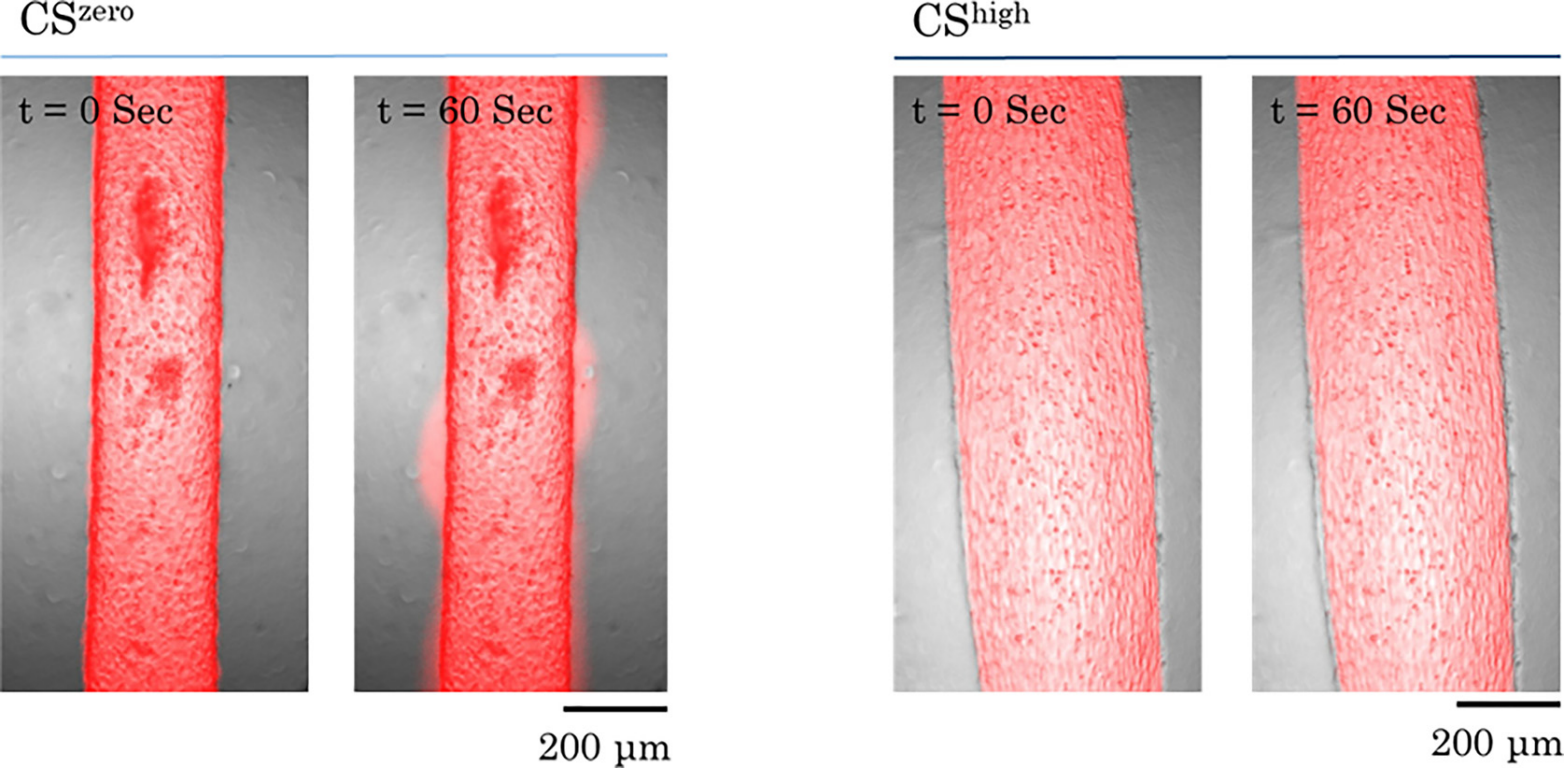
Effect of cyclic stretch on vascular permeability. Representative images of red fluorescent dye (70 kDa RITC) leakage from the vasculature wall into the fibrin matrix. Vasculatures formed by HUVECs were exposed to CS^high^ for 24 h. Under static conditions, after 60 s, fluorescent dye leaks out of the vasculature. However, high magnitudes of cyclic stretch inhibit dye leakage from the vasculature wall.

**FIG. 8.**
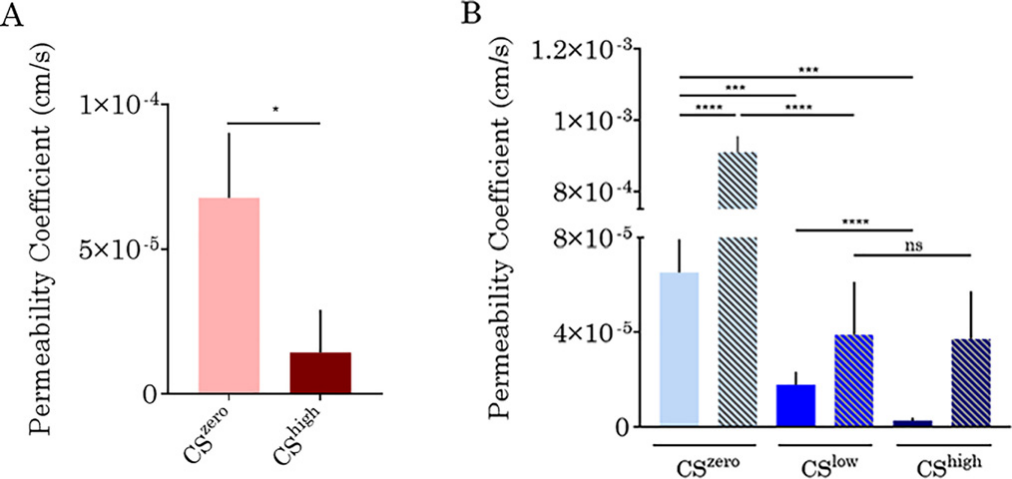
The effect of CS on vasculature formed by VeraVecs (a) and HUVECs (b). The permeability was quantified using Fiji software based on the intensity of the fluorescent dye around the vasculature. Also, for vasculature formed by HUVECs, the effect of treatment with 50 ng/ml of VEGF and interplay of cyclic stretch and treatment with VEGF on vascular permeability were investigated.

The effect of CS on the permeability of the *in vitro* vasculatures formed by VeraVecs and HUVECs was investigated. Figure 7 shows the representative images of the leakage from the vasculature wall upon CS^zero^ and CS^high^. Under static conditions, the fluorescent dye leaks into the fibrin matrix within tens of seconds. However, CS^high^ decreases leakage of the fluorescent dye [70 kDa RITC (rhodamine isothiocyanate)] into the surrounding matrix. The value of the permeability coefficient reflects the leakage level of the vascular barrier. Our results show that the leakage of the fluorescent dye (70 kDa RITC) from the endothelial barrier formed by VeraVecs reduces significantly upon CS^high^ [Fig. 8(a)].

Similarly, the exposure of the HUVEC vasculature to CS increased vascular integrity and reduced leakage from the vascular wall significantly [Fig. 8(b)]. Without VEGF treatment, CS significantly decreased the endothelial barrier permeability. Besides, the decrease in barrier permeability was also significant between CS^low^ and CS^high^, with the tighter wall that was obtained at high CS. VEGF treatment (50 ng/ml) induced a noticeable vascular leakage, but concurrent exposure to CS^low^ normalized the vascular wall integrity and decreased the permeability coefficient of the vessel. However, the vascular permeability coefficients did not show any significant difference between the two conditions involving VEGF and different magnitudes of CS [Fig. 8(b)]. The negative control was the cylindrical cavity without cells. The permeability coefficient across cylindrical cavity's wall under static and stretched conditions was compared. The leakage of the fluorescent dye from the cylinder wall encapsulated by fibrin gel did not change upon cyclic stretch.

### Cyclic stretch suppresses vascular sprouting

**FIG. 9.**
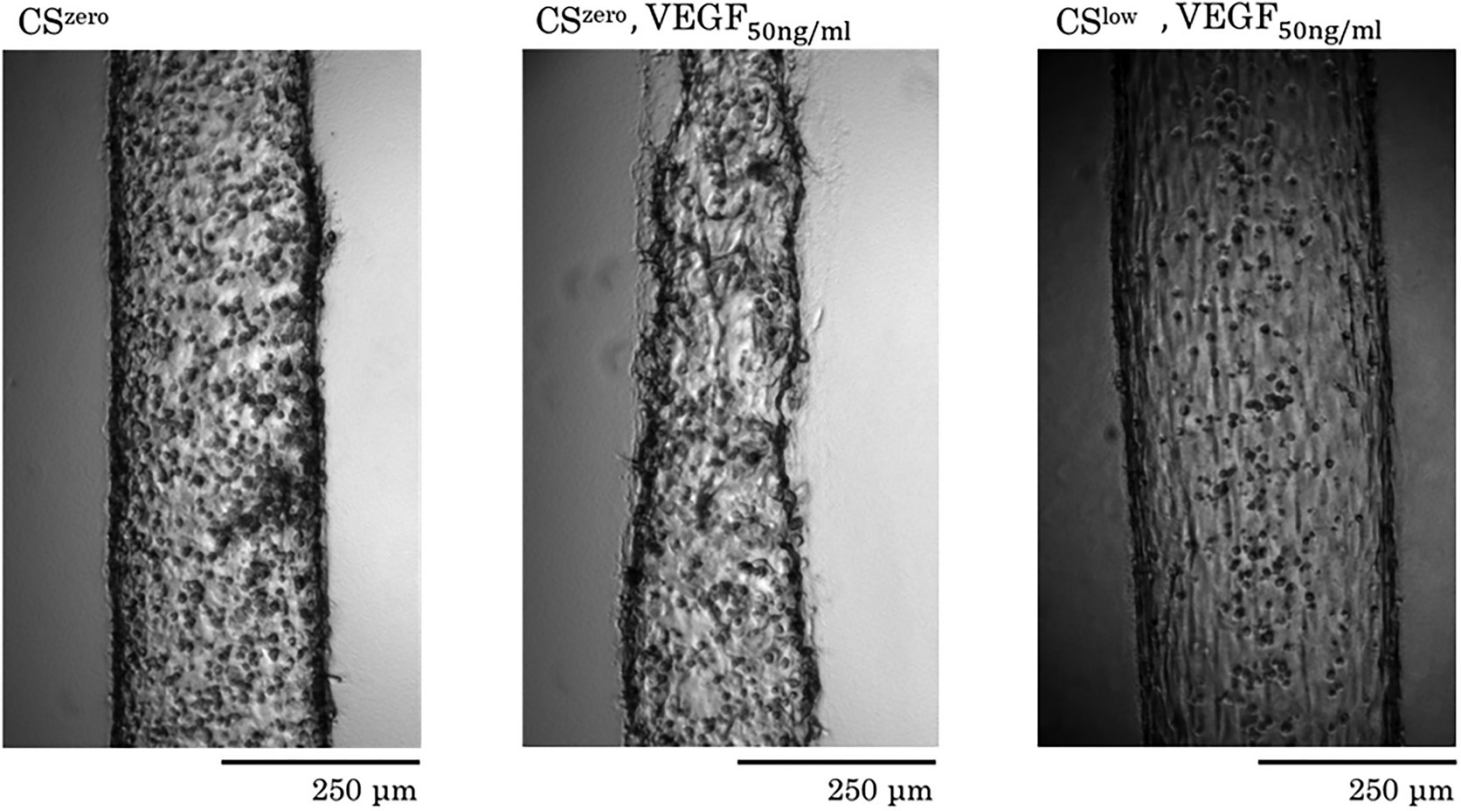
Representative images for the effects of the CS and treatment with VEGF on endothelial sprouting. Vasculature formed by HUVECs. A higher concentration of VEGF stimulates endothelial cells and increases angiogenic sprouting from parent vasculature. Mechanical cyclic stretch normalized vascular barrier and suppresses endothelial sprouting.

**FIG. 10.**
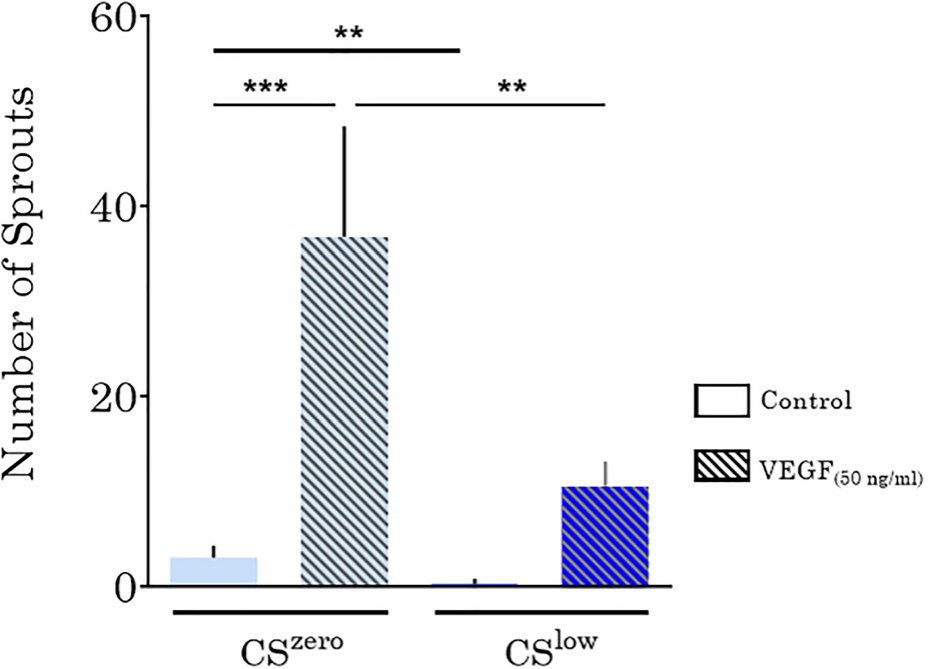
The effects of CS and VEGF (50 ng/ml) on endothelial sprouting from vasculature formed by HUVECs. CS significantly decreased endothelial sprouting from the vessel toward the surrounding tissue (fibrin gel).

Next, we examined the effects of CS and VEGF (50 ng/ml) on the endothelial sprouting of the vessel formed by HUVECs. Under the CS^zero^ condition, sprouting from the vessel toward the surrounding tissue (fibrin gel) was observed. VEGF substantially prompted sprouting from the HUVEC vasculature toward the surrounding gel matrix (Fig. 9). Under control and VEGF-treated conditions, exposure to CS^low^ for 24 h significantly reduced sprouting from the vessel toward fibrin gel around the tissue (Fig. 10). These findings indicate that, in addition to regulating the integrity and stability of the endothelial barrier, CS suppressed vascular sprouting.

### Cyclic stretch re-orients the cell nucleus

**FIG. 11.**
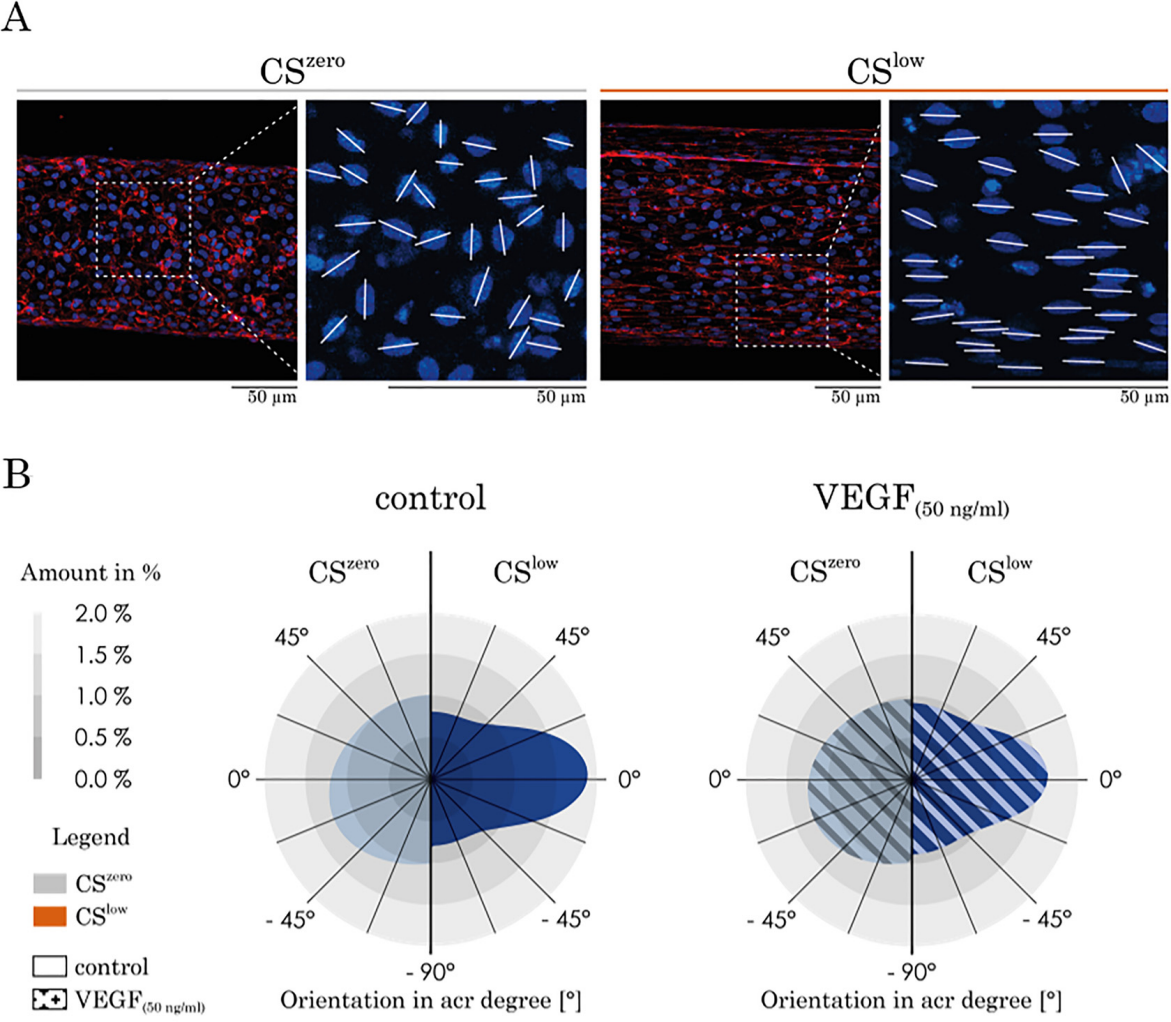
The effect of CS and VEGF on nucleus re-orientation. (a) Representative images of the HUVECs' nucleus re-orientation in CS^zero^ and CS^low^ conditions. The angles between the white lines and the horizontal line show the orientation of the nuclei. (b) Under CS^zero^, regardless of the treatment with VEGF, HUVEC nuclei are randomly oriented on the vasculature wall. However, cyclic stretch significantly re-orients the majority of cells' nuclei, leading to orientation between −20° and +20° related to the horizontal line (direction of the vasculature length).

Under static conditions, the nuclei of HUVECs are randomly oriented within the vascular wall [Fig. 11(a)]. Following exposure of the vessel formed by HUVECs to CS^low^, the nuclei were re-oriented and took the angles between −20° and +20° relative to the horizontal reference line [Fig. 11(b)]. These findings indicate that, in addition to aligning the HUVEC cytoskeleton in the direction of the vessel, 3D mechanical CS systemically orients cell nuclei in treated (VEGF) and untreated (control) conditions.

## DISCUSSION AND CONCLUSION

Vascular barrier properties highly depend on the endothelial cell response triggered by a variety of biochemical and/or mechanical stimuli. In the lungs, mechanical cyclic strains induced by breathing motions can result in dramatic vessel remodeling and changes in the vascular barrier functions.^4,45^ They are caused by cytoskeleton reorganization and alterations of adherence junctions, which lead to cellular surface area changes, loss of cell–cell contacts, and increased vascular permeability. So far, most of our knowledge about the effects of the cyclic mechanical stretch on endothelial cells is limited to *in vitro* studies using a monolayer of endothelial cells cultured on thick stretchable silicone rubber foils.^5,7,37,38^ Although a recent study investigated the simultaneous effects of the shear stress and cyclic stretch on *in vitro* vasculature, the setup could not expose the vasculature to longitudinal and circumferential strain as *in vivo*.^43^ Here, we present a functional organotypic human vasculature model that allows studying the effects of 3D mechanical cyclic stretch (simultaneous circumferential and longitudinal strains) on vascular remodeling and endothelial cell morphology.

Vasculatures formed by two different types of endothelial cells were studied: human lung microvascular endothelial cells (VeraVec) and human umbilical vein endothelial cells (HUVEC). In addition to the standard endothelial cell model, HUVECs and VeraVecs were tested for their response to 3D mechanical stretch. Vasculatures formed by both cell types respond to 3D cyclic stretch by changing the cellular morphology and the vascular barrier properties. In this study, VeraVecs were used to show that they sense and respond to 3D mechanical stimuli as three-dimensional vessels similar to HUVECs. HUVECs were further used to study more detailed effects of the vascular remodeling induced by 3D cyclic stretch and VEGF stimulation.

Our study suggests that an important cytoskeletal remodeling takes place in response to 3D CS. In both, VeraVecs and HUVECs, CS significantly increased the cell surface area, and it elongated and aligned cells in the direction of the vessel (perpendicular to longitudinal stress).

In our dynamic vasculature platform *in vitro*, vasculatures were exposed to simultaneous longitudinal and circumferential strains similar to *in vivo*. The 3D vessel underwent longitudinal and circumferential strains, with the circumferential strain level being about four times larger than that of the longitudinal strain at both low and high strain levels. These results match very well clinical data obtained by cardiac-gated computed tomography.^46^ The cyclic circumferential strain of the human thoracic aorta was found to be four times larger than the longitudinal one.^46^

Another important finding is related to the morphological response of the cells to the cyclic stretch in the form of the modification of the cell surface area. Under CS^low^, the increase in the cell surface area is not significant, unlike at CS^high^. According to Tschumperlin *et al.*,^47^ the average increase in the cell surface area under CS^low^ corresponds to a physiological strain and the average increase in the cell surface area under CS^high^ corresponds to a pathological strain. However, in sharp contrast to Birukov *et al.*,^38^ who observed endothelial gap formation and vascular wall damage at a high strain level (18% linear strain), we found in our 3D vascular model that a similar strain level shows a supportive effect on the vascular stability and barrier integrity. These findings are of high importance, as the cyclic strain levels used for mechanobiological studies of the alveolar epithelium^47^ have become the standard for *in vitro* studies investigating the effect of mechanical stretch on the endothelial barrier. According to Tschumperlin *et al.*,^3,47^ the average increase in the cell surface area under CS^low^ corresponds to a linear strain of 8%–12% and the average increase in the cell surface area under CS^high^ corresponds to a linear strain of 17%–22%. In sharp contrast, in a 3D vessel, the increase in the cell surface area under CS^high^ is 43% (HUVEC) and even 67% for lung endothelial vascular cells (Table I).

**TABLE I.** Summary of the average increase in the cell surface area and the corresponding linear strain level.

	*CS^low^*	*CS^high^*
Average increase in the surface area of HUVECs	(22 ± 1.3) %	(43 ± 1.5) %
Average increase in the surface area of VeraVecs	…	(67 ± 1.8) %
Corresponding linear strain level (based on calculations from Tschumperlin *et al.*^47^)	8%–12%	17%–22%

Importantly, these high levels of strain did not lead to a dysfunction of the endothelial barrier. In contrast, at a higher cyclic stretch level (CS^high^), the barrier stability and integrity were significantly improved compared to CS^low^. This suggests the importance of recapitulating the three-dimensional aspect of endothelial vessels. Therefore, the adoption of epithelial stretch level values for endothelial cell studies must be associated with great care.^48^

Endothelial cells (both HUVECs and Veravecs) cultured under static conditions exhibit a polygonal shape and are randomly orientated. However, CS induces a significant elongation and re-orientation of the cells in a longitudinal direction and an increase in the cell surface area. Endothelial cells elongate and align in the direction of the minimal vascular deformation (longitudinal) to minimize the mechanical tension.^45^ Interestingly, the CS induces a cell alignment regardless of VEGF treatment. Wilkins *et al.* illustrated that endothelial cells, either VEGF stimulated or not, on a 2D silicone membrane stretched uniaxially would align perpendicular to the strain direction. Our measurements of the vessel deformation subjected to CS showed that the circumferential deformation of the vessel is larger than the longitudinal deformation. Therefore, endothelial cells experience more circumferential tension and, consequently, elongate in the longitudinal direction. Similarly, endothelial cells tend to increase the cell surface area to minimize the amount of stress exerted by the cyclic deflection.

In our 3D vasculature model, treatment with VEGF increased 3D angiogenic sprouting. The angiogenic sprouting decreases, however, significantly in the presence of CS in all VEGF experiments. Yung *et al.* proposed that the combination of cyclic uniaxial strain (7%) and VEGF (10 ng/ml) on HUVEC-coated microcarriers in a fibrin gel increases sprouting angiogenesis.^49^ Wilkins *et al.* reported that a combination of CS and VEGF did not have a synergistic effect on endothelial angiogenesis but showed that CS alone induced endothelial sprouting. In our study, we did not observe CS-induced angiogenesis neither with VEGF treatment nor with CS alone. Whether the differences between the reported results and our findings are due to the different stretch levels, VEGF concentration, cell type, or angiogenesis assay remains to be elucidated. *In vivo*, however, the onset of breathing after birth triggers both CS and flow-mediated shear stress in the pulmonary vasculature, i.e., two mechanical signals that are considered critical for maturation. It is tempting to speculate that our findings of reduced sprouting upon CS resemble a stabilizing function of the mechanical stimulation exerted on blood vessels.

A 50 ng/ml VEGF treatment loosens cell–cell contacts and strikingly increases vascular permeability. Independent of the treatment, CS restores vascular barrier integrity and significantly decreases vascular permeability. Birukova *et al.* assessed endothelial permeability by analysis of morphological changes and measurements of transendothelial electrical resistance and claimed that the monolayer of endothelial cells exposed to 18% CS for 2 h to 96 h increased thrombin-induced permeability.^5^ In this study, we did not observe CS-induced leakage on the vascular barrier. In sharp contrast, we observed that CS strikingly restores the endothelial barrier integrity altered by VEGF.

To the best of our knowledge, this study is the first to investigate the interplay of VEGF and 3D CS on 3D perfusable vasculature concerning vascular morphology, angiogenic sprouting, and vascular barrier integrity. We showed that endothelial cells respond differently when exposed to different magnitudes of CS. Furthermore, regardless of the treatment with VEGF, CS increases endothelial barrier integrity, decreases angiogenic sprouting, reorients the cells, and decreases vascular barrier leakage. Advanced microvasculature-on-chip models need to mimic the *in vivo* environment, including the crucial mechanical and biochemical cues. Our *in vitro* dynamic vasculature platform represents a powerful method that allows the investigation of CS effects on real vessels and enables the identification of the role and function of individual parameters of the cell culture that is not possible in animal models. This study, however, is subjected to some potential limitations to closely mimic physiology of the blood vessels *in vitro*. Some of these limitations are lack of mural cells like pericytes besides endothelial cell, continuous flow, and consequently shear stress in the vessel, various durations and frequencies of the applied cyclic stretch, gene expression analysis, and whole blood perfusion. However, the presented dynamic vasculature platform has very high potential in investigating these limitations in future studies. In addition, such experimental platforms can address mechanistic questions of mechanotransduction and might be used for developing and optimizing drug treatment in personalized vasculature models in the future. This is a significant milestone in vascular endothelium research, as organ-level information, such as vessel remodeling and functions, can for the first time be acquired from an *in vitro* model and might one day be correlated with clinical data.

## METHODS

### Device design and fabrication

The top layer of the chip had three main compartments: a 3 mm diameter central circular chamber for microvessel generation and two reservoirs for cell seeding and medium addition connected to the central chamber through two microchannels. The bottom layer of the chip had a 3.5 mm diameter circular chamber connected to the access port of the breather through a microchannel. The two layers were separated by a 100 *μ*m thick membrane. Stereolithography (PROFORM, Fribourg, Switzerland) was used to fabricate the molds of the top and bottom layers. Polydimethylsiloxane (Sylgard, Dow Corning, USA) was mixed at a 10:1 ratio with a curing agent, cast on the mold, and cured at 65 °C overnight. To fabricate the 100 *μ*m thick membrane, the mixture of polydimethylsiloxane and curing agent was spin-coated on a silicon wafer covered by a plastic sheet at 600 rpm for 60 s and cured at 65 °C overnight. After cutting and punching the pieces of different layers, to reproduce a cylindrical cavity in the middle of the gel, a 200 *μ*m diameter needle was incorporated in the central chamber of the top layer through adjusted groves. Later, the membrane was peeled from the plastic sheet and irreversibly bonded to the top layer using O_2_ plasma (Harrick Plasma, USA). Subsequently, the bottom layer was reversibly bonded to the membrane. The breather access hole was punched through all layers, and the multilayer was bonded to a microscope slide using O_2_ plasma.

### Cell culture

Primary HUVECs (Gibco, Life Technologies Corp. C-003–5C) were cultured with endothelial growth medium 2 (EGM2; Lonza). For all experiments, HUVECs were used between passages four and nine. RFP labeled primary human lung microvascular endothelial cells (VeraVec, Angiocrine Bioscience) were cultured in EGM2-MV (Lonza). The cells used are commercially available, and therefore, no ethical approval is necessary for this study. For all experiments, VeraVecs were used between passages four and seven.

### Chip seeding and maintenance

Needle-incorporated microfluidic chips were sterilized in an ozone chamber (CoolCLAVE, Genlantis USA) before hydrogel application and cell seeding. A solution of 30 mg/ml fibrinogen from bovine plasma (Sigma) in phosphate-buffered saline (PBS; Gibco, Life Technologies Corp.) was mixed with 2 U/ml bovine plasma thrombin (Sigma) in endothelial basal medium 2 (Lonza) and immediately loaded into the central chamber of the top layer using the gel seeding ports. After fibrin gel formation (15 min), the needle was removed from the gel layer and a cylindrical cavity was generated in the middle of the fibrin gel layer. For the experiments with VeraVecs, they were suspended in EGM2-MV at a final concentration of 10 × 10^6^ cells/ml, and 5 *μ*l of the cell suspension was seeded into the reproduced microchannel of each chip through the reservoirs. Similarly, HUVECs were suspended in EGM2 at a final concentration of 10 × 10^6^ cells/ml, and a 5 *μ*l aliquot of the cell suspension was seeded into the microchannel to reproduce 3D vasculature. For all experiments with VeraVecs or HUVECs, after incubation for 30 min, EGM2 or EGM2-MV was loaded into the microchannel and the reservoirs were filled. The chips were incubated at 37 °C and 5% CO_2_ in Petri dishes containing a moist tissue for humidification. The culture medium was changed every 24 h. For some chips from the experiments with HUVECs, 50 ng/ml human VEGF^165^ (Miltenyi Biotec) in EGM2 was added to the fibrin gel (from the top of the central chamber) to induce endothelial sprouting.

### Cyclic stretch

To cyclically stretch the polymeric membrane, and consequently the microvessel, at a frequency of 0.2 Hz, the platform was connected to an external electro-pneumatic setup (house-made setup: breather). The breather controlled the magnitude of the applied negative pressure and the frequency. The pressure curve was modeled as a triangular wave and different magnitudes of linear strain on the membrane. Once the confluent vascular wall was formed (day 5), the chips were connected to the breather through the adjusted port to apply a reproducible 3D cyclic strain to the cells for 24 h.

In the experiments, the negative control was a blank cylindrical cavity without cells in the middle of the fibrin gel, which was exposed to cyclic stretch. The effect of 24 h of mechanical cyclic stretch was investigated on the fibrin gel layer incorporating a cylindrical cavity.

### Establishment of a 3D stretchable microvessel

**FIG. 12.**
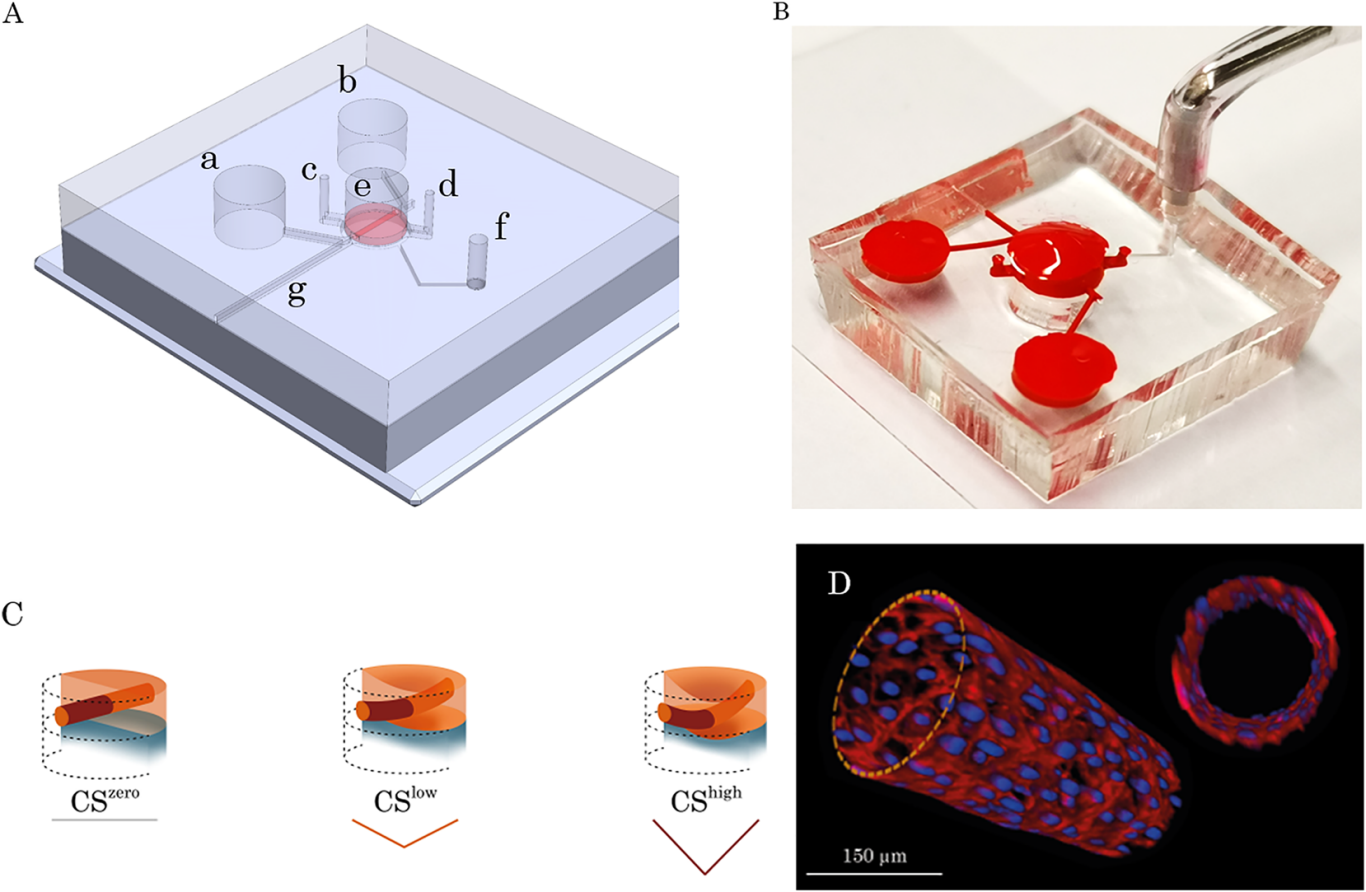
Microfluidic chip for the 3D dynamic microvasculature. (a) Schematic illustration of the multilayer dynamic microvasculature platform. The main compartments of the microfluidic chip are shown in the picture. (a) and (b) Inlet and outlet reservoirs for cell seeding and medium exchange, (c) and (d): inlet and outlet ports for fibrinogen solution loading, (e) central chamber for the fibrin gel layer incorporating a 3D vasculature, (f) access port to the bottom layer to be connected to the pump for applying negative pressure, and (g) is the groove where the needle can be inserted and removed (a small piece of tape was used to seal this groove to avoid any leakage of the fluid from the chip). (b) The PDMS platform was plasma bonded to a microscope slide and filled with red food dye for better illustration of the compartments. It comprised two main layers separated by a 100 *μ*m-thick PDMS membrane. The top layer contained a central circular chamber to insert a needle from the adjusted access port and load fibrin gel to generate a tube in the middle of the gel layer. The bottom layer had a circular chamber connected to the breather access port through a microchannel. (c) Microvasculature under different magnitudes of CS. The deflection of the membrane was achieved by applying a negative pressure from the pump, which leads to the deformation of the gel layer including the vasculature. Two different magnitudes of cyclic stretch were investigated to deflect the vasculature. (d) Illustration of the 3D microvasculature formed in the middle of the fibrin gel layer.

The design of the microfluidic chip comprises two polydimethylsiloxane (PDMS) parts, between which a 100 *μ*m thick and 3 mm diameter PDMS membrane is sandwiched. The PDMS membrane is connected on each side, with a microfluidic channel to connect two physiological medium reservoirs [Figs. 12(a) and 12(b)]. A 500 *μ*m thick fibrin layer is pipetted on the PDMS membrane inside a circular chamber incorporating a needle. After fibrinogen cross-linking and formation of the fibrin gel, the needle is gently removed through the adjusted groove and the opening was sealed using a piece of tape. Five days after seeding HUVECs in the fibrin microchannel, a 3D endothelial barrier (microvessel) was formed [Fig. 12(d)]. Fibrin gel loading, needle removal, and cell seeding in the chip processes were illustrated in an animation provided in the supplementary material (supplementary material, Video 1). The microvessel was connected to two reservoirs through two microchannels, aimed at providing nutrients and reagents [Fig. 12(a)].

### Immunostaining

A standard immunofluorescence protocol was used to stain the cells. Briefly, the cells were washed with PBS and fixed with 4% paraformaldehyde (Sigma-Aldrich) in PBS for 10 min at room temperature. After three washes with PBS, 0.1% Triton X-100 (Sigma-Aldrich) in PBS was added for 10 min to permeabilize the cells and then washed away with PBS. After blocking with 2% bovine serum albumin (BSA) in PBS for 30 min, the cells were incubated overnight with a primary antibody against the endothelial marker PECAM-1 (Cell Signaling), diluted 1:200 in PBS containing 2% BSA. Subsequently, the cells were rinsed three times with PBS and then incubated with secondary antibodies conjugated with 488 Alexa Fluor (Molecular Probes), as well as phalloidin (1:100; Invitrogen) and Hoechst (1:1000; Invitrogen), for 2 h at room temperature. After several washes with PBS, images were collected using a Zeiss LSM 710 confocal laser scanning microscope (Carl Zeiss Microscopy, LLC).

### Microvessel permeability quantification

To evaluate the effect of mechanical CS on vasculature wall integrity, the vascular permeability coefficient was quantified as described previously.^41^ At the end of day 6, the culture medium was removed from the microvessel and reservoirs, and 70 kDa rhodamine isothiocyanate-conjugated dextran (RITC, Sigma) was loaded into one of the reservoirs at a concentration of 1 mg/ml (diluted in PBS). Imaging was started before dextran loading, and images were acquired every 10 s using a Leica DMI 4000 microscope. The microvascular permeability coefficient was calculated based on the intensity of the fluorescence dye across the vascular wall over time using Fiji and the following equation:

$$P_{\mathrm{coeff}}=\frac{1}{\Delta I}\frac{dI}{dt}\frac{r}{2},$$

where ΔI is the initial intensity increase when RITC is loaded, dI/dt the change in intensity of the fluorescent dye over time due to leakage from the vessel wall, and r/2 the volume to area ratio for a cylindrical structure (2r is the vasculature diameter). Three chips per condition were quantified, and the whole region around the vessel was measured per chip.

### Cellular orientation quantification

Z-stacks of the cellular nuclei obtained from confocal microscopy images of the whole vessel were divided into two and projected on a plane with maximum signal intensity using Fiji image analysis software to give images of the top and bottom side of each vessel.^50^ These images were analyzed using the Directionality plugin in Fiji to obtain a histogram,, indicating the orientation of the cells on the vessel wall under static and dynamic conditions.

### Measurement of the cell length and surface area

Immunofluorescence images of the vasculatures obtained fluorescence microscopy was used to measure the cell length and surface area. The cell length reflects the largest diagonal length that the cell has. It was measured by using a straight-line tool in Fiji, drawing a diagonal line on the vessel, and measuring the length of the line. To measure the cell surface area, the freehand selection tool in Fiji was used to draw the boundaries around each cell and the surface area was measured. For each vessel, 100 cells were randomly chosen for measuring the cell length and surface area. The average of these cell lengths and surfaces was considered as the average length and area of the cells for that specific vessels and was repeated for three different vessels.

### Statistical analysis and data presentation

All data are represented as the mean ± standard deviation. Statistically significant differences between groups were analyzed using unpaired t-tests, and *P* < 0.05 was considered statistically significant. For each quantification and statistical analysis, the minimum number of biological samples is three (n ≥ 3). For each condition, at least three different experiments on different days with a minimum number of two chips were performed.

## SUPPLEMENTARY MATERIAL

See the supplementary material for the finite element simulation of the vasculature exposed to mechanical stretch. In supplementary material Video 1, fibrin gel loading, needle removal, and cell seeding in the chip processes are illustrated.

## Data Availability

The data that support the findings of this study are available from the corresponding author upon reasonable request.
